# Lower serum chromogranin B level is associated with type 1 diabetes and with type 2 diabetes patients with intensive conservative insulin treatment

**DOI:** 10.1186/s13098-020-00569-5

**Published:** 2020-07-14

**Authors:** Zoltan Herold, Magdolna Herold, Klara Rosta, Marton Doleschall, Aniko Somogyi

**Affiliations:** 1 Department of Internal Medicine and Hematology, Semmelweis University, H-1088 Szentkiralyi u. 46, Budapest, Hungary; 2 Department of Obstetrics and Gynecology, Medical University of Vienna, Vienna, Austria; 3 Molecular Medicine Research Group, Eotvos Lorand Research Network and Semmelweis University, Budapest, Hungary

**Keywords:** Chromogranin A, Chromogranin B, Diabetes mellitus Type 1, Diabetes mellitus Type 2

## Abstract

**Background:**

Chromogranin B (CgB) plays an important role in the physiological insulin secretion of pancreatic beta cells. Serum CgB levels were investigated in type 1 and type 2 diabetes patients in a cross-sectional study.

**Methods:**

An observational cross-sectional study was performed with the inclusion of 94 control subjects, 100 type 1 and 100 type 2 diabetes patients, at the Metabolic Outpatient Clinic of the Department of Internal Medicine and Hematology, Semmelweis University. Serum CgB levels were measured with enzyme-linked immunosorbent assay.

**Results:**

Serum CgB level was lower in type 1 diabetes patients than in matched control subjects (*p* = 0.0241), while they were equal in type 2 diabetes patients and controls (*p* = 0.1698). The subgroup of type 2 diabetes patients who received intensive conservative insulin treatment had significantly lower CgB levels compared to those with other regimens of antidiabetic therapies (*p* = 0.0283).

**Conclusion:**

The lower serum CgB levels in the patients with type 1 diabetes and the type 2 diabetes patients with progressed disease stage suggested that the CgB production might be decreased due to the beta cell destruction and depletion.

## Background

Chromogranin A (CgA) and chromogranin B (CgB) are family members of the granin glycoproteins. Both proteins are expressed by neurons, endocrine and neuroendocrine cells throughout the body in different quantities [1]. Serum CgA is routinely used as a biomarker for neuroendocrine tumors [2]. Serum levels of CgB also rise in pancreatic neuroendocrine tumors, however, it is less commonly used as a diagnostic tool [3]. Measurement of CgB can be advantageous in patients with impaired renal function and/or undergoing antacid therapy, which both may result in false positive CgA concentrations [3, 4].

Chromogranins are associated with the carbohydrate metabolism: CgA is known to play a significant role in the development and pathogenesis [5–7] of type 1 diabetes and it is associated with some type 1 diabetes complications, which could develop into neuroendocrine tumors [8]. Animal and cellular models have shown that CgB is mostly related to the physiological insulin secretion [9–11], however, human clinical study investigating the connection between CgB and diabetes has not been conducted yet.

## Methods

### Patients and study design

An observational cross-sectional study was conducted. A total of 294 study subjects with Caucasian ancestry, who attended the Metabolic Outpatient Clinic of the Department of Internal Medicine and Hematology, Semmelweis University were enrolled in the study. Written informed consent was collected from all study subjects. The study was approved by the Regional and Institutional Committee of Science and Research Ethics, Semmelweis University. The study population consisted of 100 type 1 and 100 type 2 diabetes patients, and 94 control subjects. Exclusion criteria included known tumors, inflammatory bowel disease and systemic autoimmune diseases. At the time of diagnosis, classification of diabetes was based on C-peptide levels, islet-cell and/or glutamic acid decarboxylase antibody-positivity [12]. Intensive conservative insulin treatment (ICT) for type 2 diabetes patients was defined as a multicomponent regimen including basal- and prandial insulins [13, 14].

### Clinical data and measurements

Anamnestic data and body mass index (BMI) were collected; blood samples were drawn after an 8 h fasting period. Complete blood count, glycated hemoglobin (HbA_1C_) and creatinine were measured at the Central Laboratory of Semmelweis University. Estimated glomerular filtration rate (eGFR) was calculated using the CKD-EPI equations [15]. Serum CgB levels were measured with the Human Chromogranin B (CHGB) enzyme-linked immunosorbent assay kit (dilution 10:1, abx151068, Abbexa Ltd., Cambridge, UK). Serum CgA levels were measured with the CGA-RIACT radioimmunoassay kit (CISbio International, Gif-sur-Yvette, France). As per manufacturer descriptions, both CgA and CgB kits detect larger protein sections, where no known cleavage products are located.

### Statistical analysis

Statistical analyses were performed with R version 3.6.1 [16]. Welch two-sample t-tests, Fisher’s exact tests, Spearman's rank correlation, regression models and propensity score matching (R package Matching) was used. Results are expressed as mean ± standard deviation; *p* < 0.05 was considered as statistically significant.

## Results

To test whether other factors such as age, sex, HbA_1C_, eGFR, BMI, the duration of diabetes, antacid therapy and/or known comorbidities affect serum CgB levels, univariate and multivariate regression models were used. None of these factors showed any effect on or association with CgB levels, neither within the individual study groups, nor in all of the patients. From the total of 94 control subjects, 62 and 47 age and sex matched subjects were selected via propensity score matching technique as the control population for type 1 and type 2 diabetes patients, respectively. Analysis on CgA was performed only on data where no CgA influencing factor was present [3, 4, 7]. Laboratory and anamnestic data of study subjects are summarized in Tables 1 and 2.

**Table 1:** Anamnestic and laboratory measurement data of type 1 diabetes patients

Variable	Type 1 diabetes patients [n = 100]	Age and sex matched controls [n = 62]	*p* value
Age [years]	42.2 ± 13.4	44.1 ± 16.8	0.4443
Duration of diabetes [years]	17.3 ± 10.1	–	–
Chromogranin A [ng/mL]^a^	61.64 ± 55.27	48.03 ± 19.99	0.0348
Chromogranin B [ng/mL]	89.39 ± 34.23	107.38 ± 59.77	0.0241
HbA_1C_ [%]	8.0 ± 1.7	–	–
HbA_1C_ [mmol/mol]	64.0 ± 18.6	–	–
White blood cell count [10^9^/L]	6.91 ± 1.77	7.08 ± 1.95	0.5894
Red blood cell count [10^12^/L]	4.88 ± 0.51	5.04 ± 0.56	0.0739
eGFR [mLmin^−1^(1.73m^2^)^−1^]	100.79 ± 18.65	100.26 ± 16.10	0.8483
Body mass index (BMI) [kg/m^2^]	25.6 ± 5.0	25.9 ± 5.3	0.7505
Sex (Female / Male)	50: 50	31: 31	1.0000
Hypertension	47	17	0.0140
Thyroid disease	31	4	0.0002
Gastroesophageal reflux disease	14	7	0.8104
Antacid therapy	6	5	0.7499

Values are expressed as mean ± standard deviation, unit of anamnestic data is the number of observations

*eGFR* Estimated glomerular filtration rate, *HbA*_*1C*_ Glycated hemoglobin

^a^Excluding the measurement of study subjects, who had any condition leading to chromogranin A elevation [3, 4, 7]. Remaining number of observations was 91 and 56, respectively

**Table 2:** Anamnestic and laboratory measurement data of type 2 diabetes patients

Variable	Type 2 diabetes patients [n = 100]	Age and sex matched controls [n = 47]	*p* value
Age [years]	63.0 ± 11.6	58.4 ± 14.6	0.0589
Duration of diabetes [years]	13.7 ± 10.3	–	–
Chromogranin A [ng/mL] ^a^	57.80 ± 34.74	49.97 ± 22.29	0.1587
Chromogranin B [ng/mL]	99.72 ± 54.79	112.54 ± 61.68	0.1698
HbA_1C_ [%]	7.3 ± 1.2	–	–
HbA_1C_ [mmol/mol]	56.0 ± 13.1	–	–
White blood cell count [10^9^/L]	7.93 ± 2.08	7.21 ± 1.91	0.0443
Red blood cell count [10^12^/L]	4.82 ± 0.39	5.01 ± 0.56	0.0389
eGFR [mLmin^−1^(1.73m^2^)^−1^]	83.49 ± 17.84	90.19 ± 13.13	0.0117
Body mass index (BMI) [kg/m^2^]	31.1 ± 5.8	27.9 ± 5.4	0.0017
Sex (Female / Male)	50: 50	24: 23	1.0000
Hypertension	91	17	< 0.0001
Thyroid disease	16	4	0.3038
Gastroesophageal reflux disease	31	5	0.0074
Antacid therapy	29	3	0.0013

Values are expressed as mean ± standard deviation, unit of anamnestic data is the number of observations

*eGFR* Estimated glomerular filtration rate, *HbA*_*1C*_ Glycated hemoglobin

^a^Excluding the measurement of study subjects, who had any condition leading to chromogranin A elevation [3, 4, 7]. Remaining number of observations was 64 and 43, respectively

**Fig. 1:**
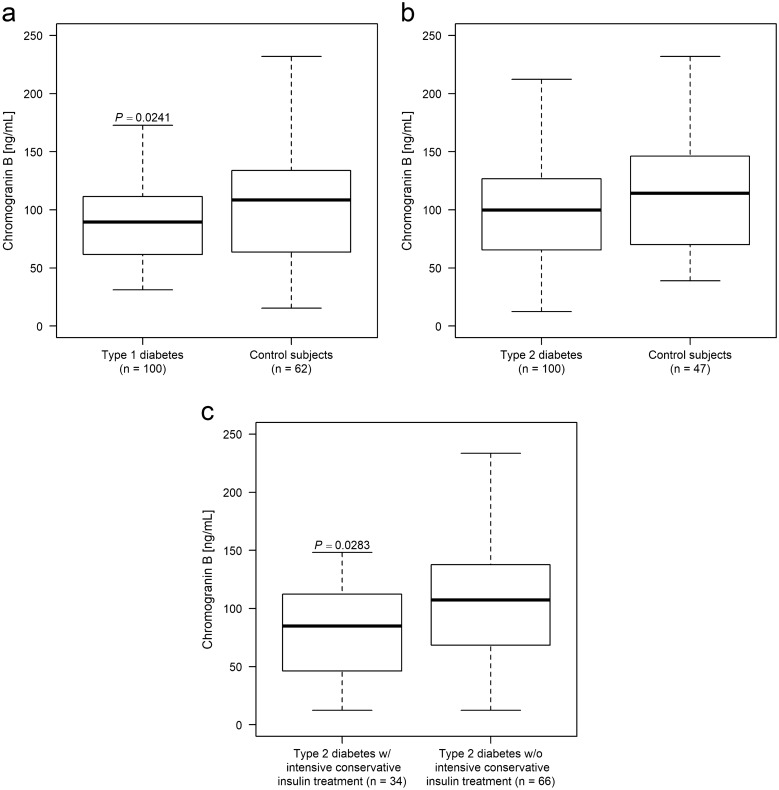
Serum chromogranin B (CgB) level of study subjects. In type 1 diabetes patients serum CgB levels were significantly lower than those in sex- and age-matched controls (**a**), whereas in type 2 diabetes (**b**) the 2 cohorts did not differ. Subgroup analysis depending on the type of therapy in type 2 diabetes (**c**) revealed that patients on intensive conservative insulin treatment had significantly lower serum CgB levels, compared to other regimens of therapy

Serum CgA level was significantly higher (*p* = 0.0348) in type 1 diabetes patients than that of healthy controls (Table 1), in accordance with our previous reports [8]. Type 1 diabetes patients had significantly lower serum CgB level (*p* = 0.0241) than the corresponding age- and sex matched control subjects (Fig. 1a). No correlation was found between serum CgA and CgB levels (*p* = 0.7271).

Neither serum CgB levels (*p* = 0.1698; Fig. 1b), nor CgA levels (*p* = 0.1587; Table 2) of all type 2 diabetes patients differed from those of their matched controls. The subgroup of type 2 diabetes patients with ICT (n = 34, CgB: 84.87 ± 40.37 ng/mL) have significantly lower serum CgB levels (*p* = 0.0283; Fig. 1c), compared to those patients, who are treated with any other regimens of antidiabetic therapies (n = 66, CgB: 107.38 ± 59.74 ng/mL). No correlation could be verified between serum CgA and CgB levels (*p* = 0.7635).

## Discussion

Serum CgA and CgB have higher levels in blood and correlate with each other in neuroendocrine tumors of various locations including foregut, midgut and adrenal medulla [2–4]. Interestingly, CgA and CgB levels did not correlate with each other and changed in different directions in type 1 diabetes mellitus. The serum CgA level increases with the progression of type 1 diabetes, and it is associated with the development of enterochromaffin-like cell hyperplasia and autoimmune gastritis [8], from which neuroendocrine tumors can arise [17, 18]. In contrast, serum CgB level was lower in the patients with type 1 diabetes than those in healthy controls. We have demonstrated that neither CgA nor CgB level differed in type 2 diabetes patients from those in healthy controls, but serum CgB level was lower in the subgroup of type 2 diabetes patients with ICT.

CgB derives from numerous neurons, endocrine and neuroendocrine cell types throughout the human body [1]. CgB contributes to the physiological secretion of insulin in pancreatic beta cells [9–11], moreover, it may take part in the signal transduction of insulin secretion [19]. Furthermore, the messenger RNA quantity of CgB is reduced in pancreatic islets of type 2 diabetes patients than that of non-diabetic control subjects [20]. CgB was significantly lower with 17% in type 1 diabetes patients and with 21% in type 2 diabetes patients with ICT compared to the matched groups in the current study. Both type 1 diabetes patients and type 2 diabetes patient with ICT featuring progressed disease state have got pancreatic beta cell damage to different extent [21, 22]; and the need for ICT is an indirect indication of beta cell damage [23]. Therefore, the reduced CgB production due to beta cell damage may cause the observed lower serum CgB levels in diabetes.

## Conclusions

In summary, the autoimmune destruction of beta cells might be the cause behind the lower serum CgB level in type 1 diabetes, and lower CgB were observed in type 2 diabetes with progressed disease state, which is also characterized by beta cell damage, however, this assumed cause is hardly proven in humans. Limitations of the current study are the relatively smaller sample size and the lack of serum C-peptide measurements. To support the assumed causative relationship between CgB and diabetes, further functional investigations are needed in model systems.

## Data Availability

The datasets used and/or analysed during the current study are available from the corresponding author on reasonable request.

## Abbreviations

BMI: Body mass index
CgA: Chromogranin A
CgB: Chromogranin B
eGFR: Estimated glomerular filtration rate
HbA_1C_: Glycated hemoglobin
ICT: Intensive conservative insulin treatment
